# Anti-Sm antibodies in the classification criteria of systemic lupus erythematosus

**DOI:** 10.1016/j.jtauto.2022.100155

**Published:** 2022-04-13

**Authors:** Joyce J.B.C. van Beers, Marco W.J. Schreurs

**Affiliations:** a Central Diagnostic Laboratory, Maastricht University Medical Center, Maastricht, the Netherlands; b Laboratory of Medical Immunology, Department of Immunology, Erasmus MC, University Medical Center Rotterdam, the Netherlands

**Keywords:** Systemic lupus erythematosus, Classification criteria, Smith antigen

## Abstract

Systemic lupus erythematosus is characterized by autoantibodies and immune complex deposition. Several autoantibodies against mainly nuclear autoantigens have been described. One of these nuclear autoantigens is the Smith antigen. In this review, we focus on the position of autoantibodies against the Smith antigen in the classification criteria, the characteristics of the antigen, the production of anti-Smith antibodies in SLE and we discuss the different test methods available, together with their pitfalls, to detect these autoantibodies.

## Highlights

• Patients having anti-Sm antibodies already fulfil sixty percent of the criteria required for SLE classification.
• Correct interpretation of anti-Smith antibody test results is strongly related to the choice of the test used for detection.
• Anti-Sm antibodies are very specific for SLE.

## Abbreviations

ACR: American College of Rheumatology
ALBIA: addressable laser bead immunoassay
ANA: antinuclear antibody
BAFF(R): B-cell activating factor belonging to TNF family (receptor)
BCR: B-cell receptor
CLIA: chemiluminescent immunoassay
DAMPs: danger-associated molecular patterns
ELISA: enzyme-linked immunosorbent assay
EULAR: European League Against Rheumatism
FEIA: fluorescent enzyme immunoassay
IFNAR: interferon-α/β receptor
IIFT: indirect immunofluorescence test
LAIR1: Leukocyte-associated immunoglobulin-like receptor 1
LIA: line immunoassay
LIR1: leukocyte immunoglobulin-like receptor-1
MCTD: mixed connective tissue disease
PAMPs: pathogen-associated molecular patterns
PD-1(L): programmed Cell Death (Ligand) 1
RIA: radioactive immunoassay
RNP: ribonucleoprotein
SLE: Systemic lupus erythematosus
SLICC: Systemic Lupus International Collaborating Clinics
SnRNA: Small nuclear RNA
snRNP: small nuclear ribonucleoprotein
TLR: Toll-like receptor

## 1 Introduction

Systemic lupus erythematosus (SLE) is a chronic, systemic rheumatic autoimmune disease, which is characterized by autoantibodies and immune complex deposition, which can basically affect any organ leading to a range of clinical manifestations. Besides clinical manifestations, serological findings play an important role in diagnosis. Because diagnostic criteria are lacking, classification criteria, which are primarily designed for including patients for clinical studies, are often used for diagnostic purposes. In 1971, the first classification criteria for SLE were described, which were first revised in 1982. These revised criteria included, amongst fluorescence antinuclear antibody (ANA) and antibody to native DNA, antibody against the Smith (Sm) antigen, which improved the performance of the criteria [1]. These antibodies, including antiphospholipid autoantibodies, which have been incorporated in the criteria since 1982, are nowadays still part of the SLE classification criteria, The European Alliance of Associations for Rheumatology (EULAR; formerly known as European League Against Rheumatism)/American College of Rheumatology (ACR) classification criteria, as well as the Systemic Lupus International Collaborating Clinics (SLICC) criteria. Besides the presence of autoantibodies, the SLICC criteria comprises additional serological criteria, such as low complement levels (C3, C4 or CH50) and a positive direct Coombs test in the absence of haemolytic anaemia [[2], [3], [4]].

In the 2019 EULAR/ACR criteria, ANA, tested by immunofluorescence on HEp-2 cells or an equivalent solid-phase ANA screening immunoassay, was introduced as an entry criterion hereby excluding ANA negative patients to be classified as having SLE [2,5]. In addition, in the 2019 EULAR/ACR criteria, within both clinical and immunology domains different criteria have been appointed different weights. Antibodies against the Smith (Sm) antigen have been assigned 6 points within the immunology domain, which is more than half of the total score required for SLE classification [2].

## 2 The Smith antigen

The name Smith antigen is derived from a patient named Stephanie Smith, who was diagnosed with SLE in 1959. Her physician dr. Tan, discovered a specific SLE antigen using the Ouchterlony agar diffusion method with her serum. This specific SLE antigen became known as the Smith antigen or Sm antigen [6,7].

The Sm antigen represents not a single protein but a protein complex consisting of a group of core proteins. So far several proteins, being SmB1 (SmB), SmB2 (SmB’), SmB3 (SmN), SmD1, SmD2, SmD3, SmE, SmF and SmG, have been identified, which are expressed in the nuclei of all cells [8].

The Sm proteins, together with ribonucleoproteins and small nuclear RNA (snRNA) form a RNA-protein complex or small nuclear ribonucleoprotein (snRNP), which is involved in precursor messenger RNA (mRNA) splicing, a process which ultimately leads to mature mRNA generation [9].

**Fig. 1:**
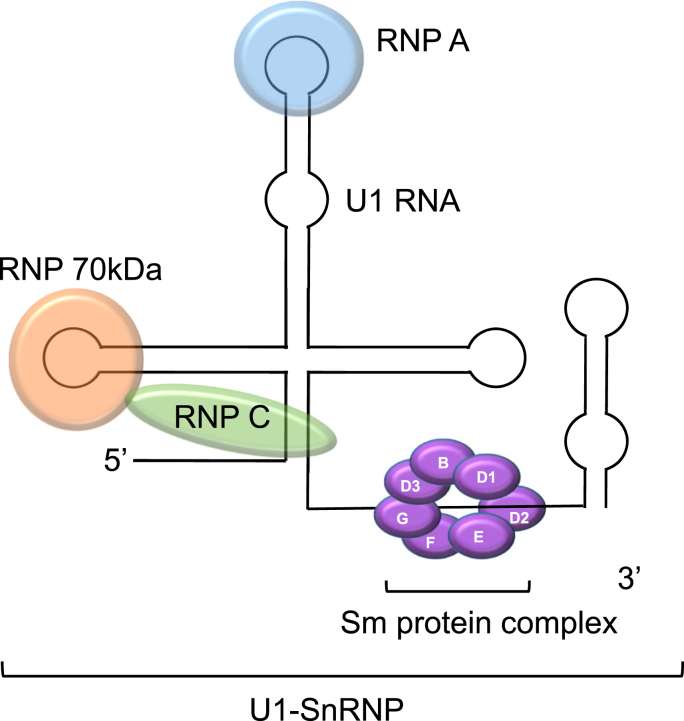
The U1 small nuclear ribonucleoprotein complex. The U1 small nuclear ribonucleoprotein (U1-snRNP) complex consists of the Sm protein complex, which includes 9 different proteins (B1, B2 and B3, D1, D2, D3, E, F and G), ribonucleoproteins (RNP 70 KDa, RNP A and RNP C) and U1 ribonucleic acid (U1 RNA). RNA: ribonucleic acid; RNP: ribonucleoproteins; Sm: Smith protein.

The Sm protein complex binds to the snRNA as a ring-liked structure, hereby protecting the snRNA from degradation by nucleases and supporting RNA-processing. There are different snRNAs known that are part of different snRNPs (e.g. U1, U2, U4/U6 and U5). U1-SnRNP is an example of a well-known spliceosome, which consists of U1-RNA, the ribonucleoproteins RNP70, A and C and Sm Proteins (Fig. 1). U1-snRNP also plays a role in RNA processing (e.g. polyadenylation). Interestingly, antibodies against U1-RNP are present in all patients with mixed connective tissue disease (MCTD), a condition that shares clinical features with SLE [10].

The Sm proteins are highly conserved in eukaryotes and share highly conserved motifs (Sm motif 1 en motif 2), which are involved in the interactions between the different Sm proteins [8,11,12].

## 3 The formation of anti-Sm antibodies in SLE patients

Many autoantibodies have been described in SLE, but only a few antibodies, including anti-dsDNA, anti-Ro/La and anti-Sm, can be detected more frequent in SLE patients [[13], [14], [15]]. Anti-Sm antibodies, which were first identified in 1966, are detected in approximately 5–30%, depending on ethnicity and detection method used to identify the antibodies. Anti-Sm antibodies are, however, very specific for SLE and are often already present prior to diagnosis [6,16]. In SLE patients anti-Sm antibodies are mainly directed to the SmB (B1, B2 and B3) and SmD (D1, D2 and D3) proteins. However, due to cross-reactive epitopes shared between U1-RNP and SmB proteins, SmD proteins, especially SmD1 and SmD3, are considered to be the most SLE specific antigens [17].

The precise mechanism of anti-Sm antibody formation in SLE patients is not fully understood.

It is known that SLE is a typical autoimmune disease, in which loss of B-cell tolerance and subsequently the formation of autoreactive B-cells and production of autoantibodies recognizing self-antigens is a key feature [18].

### 3.1 Loss of self-tolerance

**Fig. 2:**
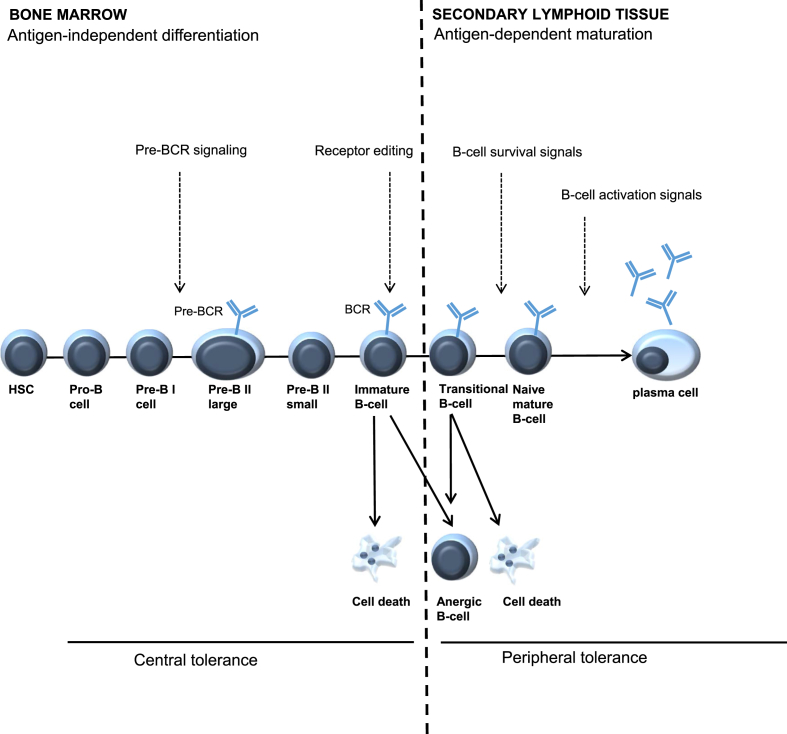
B-cell development is subjected to different checkpoints. During normal B-cell development several checkpoints (indicated by the arrows) are present to limit the loss of self-tolerance, in the bone-marrow (central tolerance) as well as in the periphery and secondary lymphoid tissue (peripheral tolerance).

To prevent autoimmunity, B-cell activation is strictly regulated. Normally, the majority of the autoreactive B-cells are deleted (e.g. by negative selection) before leaving the bone-marrow. Several self-tolerance checkpoints are present during B-cell development [19,20] (Fig. 2). Central tolerance may be obtained by clonal deletion (negative selection), anergy and B-cell receptor (BCR) editing. Despite these mechanisms, several autoreactive B-cells may escape and continue to develop in the periphery. The destiny of the B-cell is heavily dependent of BCR signalling [18]. In the periphery, autoreactive B-cells are normally removed by apoptosis due to diminished survival signals (i.e. too strong BCR signalling and/or to weak co-stimulatory signal).

In SLE patients abnormal B-cell maturation (i.e. more pre-naïve and transitional B-cells and an enlarged pool of isotype-switched memory B-cells), negative selection, receptor editing and antigen responsiveness (e.g. altered BCR signalling strength) has been observed, either resulting primarily from a B-cell defect or secondary as a result of inflammation [20,21].

In addition, it has been suggested that in SLE, the mechanisms behind the clearance of apoptotic cells, due to Fas-Fas ligand pathway defects and decreased phagocytosis are impaired. Moreover, the degradation of neutrophil extracellular traps (NETs) is defective [22]. It has been shown that the bone marrow of SLE patients consists of more apoptotic cells. This may lead to an overexposure to (nuclear) antigens, such as the Sm antigen, of the developing B-cell [23].

**Fig. 3:**
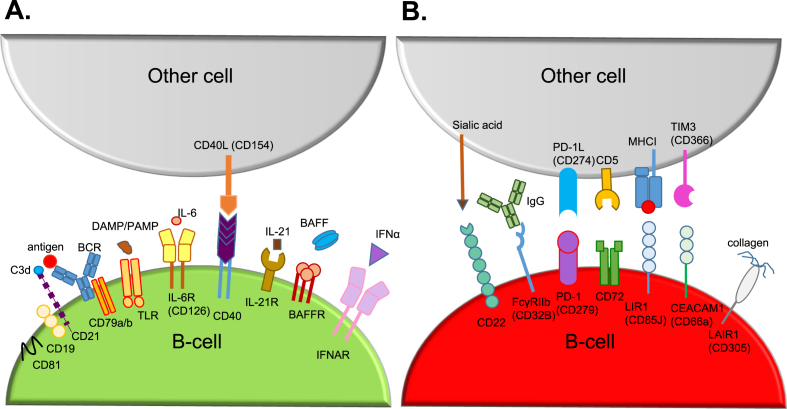
Checkpoints can either stimulate or inhibit B-cell development. B-cells express a broad range of stimulatory (A) and inhibitory (B) checkpoints, of which a selection is depicted in this figure. In SLE, different checkpoint molecules can be modulated (e.g. due to genetic modifications) leading to altered B-cell activation. BAFF(R): B-cell activating factor belonging to TNF family (receptor); BCR: B-cell receptor; DAMPs: danger-associated molecular patterns; IFNAR: interferon-α/β receptor; LAIR1: Leukocyte-associated immunoglobulin-like receptor 1; LIR1: leukocyte immunoglobulin-like receptor-1; PAMPs: pathogen-associated molecular patterns; PD-1(L): programmed cell death (Ligand) 1; TLR: Toll-like receptor.

Several stimulatory and inhibitory checkpoints have been described previously, that can modulate B-cell development and function. Examples of such checkpoints are depicted in Fig. 3 [[24], [25], [26], [27]]. Genetic abnormalities in certain molecules, that serve as a checkpoint and are important for maintaining normal B-cell development, together with environmental factors, contribute to autoantibody formation [20,24,27].

Cytokines BAFF, IL-6 and IFNα play an important role during SLE disease activity. BAFF levels are increased in SLE patients leading to excessive B-cell stimulation. While targeting IL-6 and IFNα have shown no to minimal beneficial effects on disease activity, targeting BAFF (Belimumab) is an effective treatment for antibody positive SLE patients. BAFF levels have been associated with the presence of dsDNA antibodies but also anti-Sm antibodies [28]. In addition, BAFF levels may be related to renal involvement seen in SLE patients, however, additional research is still needed to confirm this finding [29].

### 3.2 The role of anti-Sm antibodies in SLE pathogenesis

A distinctive of feature of SLE is immune complexes containing antibodies against dsDNA play a central role in pathogenesis of lupus nephritis [30,31]. Immune deposits with anti-Sm antibodies have been detected in glomeruli of SLE patients [32]. Others have identified antibodies against RNP/Sm in circulating immune complexes of SLE patients [33,34] However, the clinical significance of anti-Sm antibodies is still not clear. Some studies reported that anti-Sm antibodies are associated with different disease presentations that occur in SLE patients, such as renal involvement, neuropsychiatric manifestations, hemolytic anemia, and vasculitis, while other studies reported no association with clinical manifestations at all [35,36]. Because of these conflicting data, anti-Sm antibodies are currently not regarded suitable as a prognostic or activity marker in SLE.

Previous EBV exposure has been proposed to play a potential role in the generation of the anti-Sm response as a result of molecular mimicry due to a shared sequence between Sm proteins (SmB as well SmD) and Epstein-Barr virus encoded nuclear antigen (EBNA) [[37], [38], [39]].

## 4 Anti-Sm antibody testing: the possibilities and the pitfalls in clinical interpretation

There are various techniques that can be used to detect anti-Sm antibodies. The classification criteria do not define, which test should be used for screening for anti-Sm reactivity. The gold standard technique to detect anti-Sm antibodies is immunoprecipitation using a radioactive (e.g. S35-methionine labelled cell extract) immunoassay (RIA). In daily practice, other techniques, including indirect immunofluorescence tests (IIFT) and antigen-specific (solid-phase) immunoassays, such as enzyme-linked immunosorbent assays (ELISA), addressable laser bead immunoassays (ALBIA), line immunoassays (LIA), chemiluminescent immunoassays (CLIA) and fluorescent enzyme immunoassays (FEIA), are more widely used. These antigen specific immunoassays use, either a mixture of (native) Sm antigens or a specific (recombinant) Sm antigen, usually obtained by purification of nuclear extract or produced by in vitro translation, respectively, coated to a solid phase (e.g. plate/well, membrane, bead) [40].

**Fig. 4:**
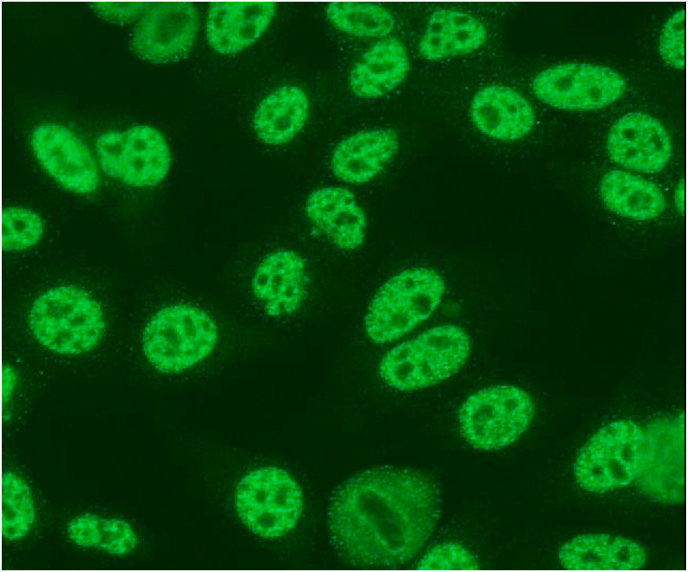
Anti-Smith antibodies give rise to a speckled ANA staining. When performing IIFT on HEp-2 cells or variants of this cell line, antibodies against the Sm antigen will lead to a nuclear (large, course) speckled pattern (AC-5) [42]. Nucleoli in the nucleoplasm may be stained or may not be stained. The chromatin is typically not stained. Note: image is taken at the laboratory of Medical Immunology of the department of Immunology at the Erasmus Medical Center in Rotterdam, The Netherlands.

Almost all SLE patients have a positive ANA. The sensitivity of the test, which can differ among the different test systems used, is overall very high. In SLE, ANA are mainly directed against nuclear components, such as DNA and histones, and RNA-binding proteins, such as the Sm antigen [41]. Antibodies against the Sm antigen will lead to a nuclear (large, course) speckled pattern (AC-5, as depicted in Fig. 4) [42,43]. This pattern, which is not specific for anti-Sm antibodies, is only observed when performing the classical IIFT on HEp-2 cells or variants of this cell line (i.e., HEp-2000) [42,44]. Nowadays, however, to detect ANA many laboratories use a solid phase assay-based screening test (connective tissue disease screen or CTD-screen), which includes a mixture of purified and/or recombinant autoantigens relevant for several systemic autoimmune rheumatic diseases, instead of using IIFT. Besides being an overall more labour-intensive method, IIFT test performance and pattern interpretation can be less consistent than fully automated solid phase assays, such as a CTD screen [45,46].

A positive result with a CTD screen assay is, however, heavily dependent on the autoantigen used as a substrate in the performed assay. If CTD screen results are reported as ANA, this may have effect on SLE classification and potentially also diagnosis, because in the latest EULAR/ACR criteria, ANA negative patients are already classified as not having SLE. Therefore, it is important for laboratories the specify the methods used for ANA detection, because different assays have different strengths and limitations for instance with respect to sensitivity and specificity [5].

**Fig. 5:**
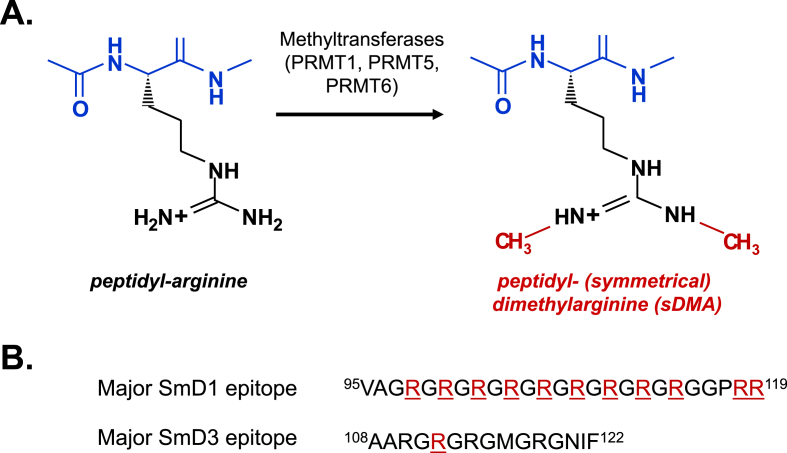
Post-translational modifications lead to SmD peptides with increased specificity for SLE. Arginine can be converted into a symmetrical dimethylarginine by methyltransferases (A). This post-translational modification leads to more SLE specific SmD1 and SmD3 epitopes (B). These epitopes range from amino acid position 95 until 119 for the SmD1 antigen and amino acid position 108 until 122 for the SmD3 antigen. Symmetrical dimethylargine residues are underlined and depicted in red. PRMT = Protein Arginine MethylTransferases.

A positive ANA, tested by either IIFT or CTD screen, should always be followed by an antigen-specific test to confirm the presence of anti-Sm antibodies. In the past anti-Sm tests used a mixture of all Sm proteins purified from a native source. These mixtures often also contained other proteins, such as U1-RNP. Nowadays, antigen specific tests mostly use either purified single antigen, in vitro translated single antigen or synthetic peptide. The antigen source can differ between different tests [40,[47], [48], [49], [50]]. As already mentioned SmD1 and SmD3 proteins are regarded to be the most specific antigen targets in SLE [51]. With techniques used for epitope mapping (e.g. by performing immunoassays with overlapping synthetic SmD1 and SmD3 peptides covering the complete protein), major SmD1 and SmD3 epitopes have been identified. Moreover, it was observed, when specific arginines present in these synthetic peptides were replaced by a symmetrical dimethylargine, binding to sera derived from SLE patients increased exceptionally [[52], [53], [54]] (Fig. 5).

Almost all the immunoassays used to detect anti-Sm antibodies focus on IgG isotype antibodies. Anti-Sm IgE, in addition to IgG, antibodies have been described before. These IgE antibodies will not be detected in assays that use an IgG specific conjugate [55].

Test accuracy, including sensitivity and specificity, not only depends on the source of antigen used, but also on how a specific antigen is coated to the solid surface used in the specific test systems. Recently, it was observed that by using the same Sm-derived peptide, but by applying a different coating technique, an increased specificity without altering sensitivity was observed [56].

Finally, comparing autoantibody test results obtained from different laboratories can be very challenging, due to the absence of harmonization between different autoantibody tests. Individual tests apply their own cut-off values, which are specified by the manufacturer of the test. An option the overcome these differences is by using test result specific likelihood ratios, as already proposed previously, rather than manufacturer's specific cut-off values [40,57].

## 5 Conclusions

Anti-Sm antibodies are an important part of the previous and current SLE classification criteria. Anti-Sm antibodies, especially antibodies directed against the SmD antigen, are very specific for SLE, however, sensitivity is restricted. The clinical significance of anti-Sm antibodies is still under debate.

Many tests are available for detecting the presence of anti-Sm antibodies. These tests have different variables (e.g. source of the Sm antigen, different coating, different cut-off values), which can affect the performance and interpretation of the test. For optimal interpretation of anti-Sm test results, that currently lack harmonization, it is important that both laboratory specialists and clinicians communicate and are both aware of the impact of the choice of anti-Sm assay and the way results are reported.

## Credit author statement

Joyce J.B.C. van Beers: writing – original draft. Marco W.J. Schreurs: writing – reviewing and editing.

## Declaration of competing interest

The authors declare that they have no known competing financial interests or personal relationships that could have appeared to influence the work reported in this paper.
